# Estimates of Bivalent mRNA Vaccine Durability in Preventing COVID-19–Associated Hospitalization and Critical Illness Among Adults with and Without Immunocompromising Conditions — VISION Network, September 2022–April 2023

**DOI:** 10.15585/mmwr.mm7221a3

**Published:** 2023-05-26

**Authors:** Ruth Link-Gelles, Zachary A. Weber, Sarah E. Reese, Amanda B. Payne, Manjusha Gaglani, Katherine Adams, Anupam B. Kharbanda, Karthik Natarajan, Malini B. DeSilva, Kristin Dascomb, Stephanie A. Irving, Nicola P. Klein, Shaun J. Grannis, Toan C. Ong, Peter J. Embi, Margaret M. Dunne, Monica Dickerson, Charlene McEvoy, Julie Arndorfer, Allison L. Naleway, Kristin Goddard, Brian E. Dixon, Eric P. Griggs, John Hansen, Nimish Valvi, Morgan Najdowski, Julius Timbol, Colin Rogerson, Bruce Fireman, William F. Fadel, Palak Patel, Caitlin S. Ray, Ryan Wiegand, Sarah Ball, Mark W. Tenforde

**Affiliations:** ^1^Coronavirus and Other Respiratory Viruses Division, National Center for Immunization and Respiratory Diseases, CDC; ^2^Westat, Rockville, Maryland; ^3^Section of Pediatric Infectious Diseases, Department of Pediatrics, Baylor Scott & White Health, Temple, Texas; ^4^Department of Medical Education, Texas A&M University College of Medicine, Temple, Texas; ^5^Influenza Division, National Center for Immunization and Respiratory Diseases, CDC; ^6^Children’s Minnesota, Minneapolis, Minnesota; ^7^Department of Biomedical Informatics, Columbia University Irving Medical Center, New York, New York; ^8^NewYork-Presbyterian Hospital, New York, New York; ^9^HealthPartners Institute, Minneapolis, Minnesota; ^10^Division of Infectious Diseases and Clinical Epidemiology, Intermountain Healthcare, Salt Lake City, Utah; ^11^Kaiser Permanente Center for Health Research, Portland, Oregon; ^12^Kaiser Permanente Vaccine Study Center, Kaiser Permanente Northern California Division of Research, Oakland, California; ^13^Center for Biomedical Informatics, Regenstrief Institute, Indianapolis, Indiana; ^14^School of Medicine, Indiana University, Indianapolis, Indiana; ^15^School of Medicine, University of Colorado Anschutz Medical Campus, Aurora, Colorado; ^16^Vanderbilt University Medical Center, Nashville, Tennessee; ^17^Fairbanks School of Public Health, Indiana University, Indianapolis, Indiana.

On September 1, 2022, CDC’s Advisory Committee on Immunization Practices (ACIP) recommended a single bivalent mRNA COVID-19 booster dose for persons aged ≥12 years who had completed at least a monovalent primary series. Early vaccine effectiveness (VE) estimates among adults aged ≥18 years showed receipt of a bivalent booster dose provided additional protection against COVID-19–associated emergency department and urgent care visits and hospitalizations compared with that in persons who had received only monovalent vaccine doses (*1*); however, insufficient time had elapsed since bivalent vaccine authorization to assess the durability of this protection. The VISION Network* assessed VE against COVID-19–associated hospitalizations by time since bivalent vaccine receipt during September 13, 2022–April 21, 2023, among adults aged ≥18 years with and without immunocompromising conditions. During the first 7–59 days after vaccination, compared with no vaccination, VE for receipt of a bivalent vaccine dose among adults aged ≥18 years was 62% (95% CI = 57%–67%) among adults without immunocompromising conditions and 28% (95% CI = 10%–42%) among adults with immunocompromising conditions. Among adults without immunocompromising conditions, VE declined to 24% (95% CI = 12%–33%) among those aged ≥18 years by 120–179 days after vaccination. VE was generally lower for adults with immunocompromising conditions. A bivalent booster dose provided the highest protection, and protection was sustained through at least 179 days against critical outcomes, including intensive care unit (ICU) admission or in-hospital death. These data support updated recommendations allowing additional optional bivalent COVID-19 vaccine doses for certain high-risk populations. All eligible persons should stay up to date with recommended COVID-19 vaccines.

The VISION Network evaluated VE of bivalent vaccines against COVID-19–associated hospitalization by length of time since receipt of the most recent dose during September 13, 2022–April 21, 2023, across five sites in seven states. VE methods used by the VISION Network have been previously described (*2*). For this analysis, adults aged ≥18 years with and without immunocompromising conditions who were hospitalized with COVID-19–like illness^†^ were included if the patient received molecular testing (e.g., real-time reverse transcription–polymerase chain reaction) for SARS-CoV-2 during the 14 days preceding or up to 72 hours after hospital admission. Patients were categorized as immunocompromised or not based on *International Classification of Diseases, Tenth Revision *(ICD-10) discharge codes.^§^ Patients were classified on the index date^¶^ as unvaccinated (no COVID-19 vaccine doses received), vaccinated with monovalent doses only, or vaccinated with one mRNA bivalent booster dose (regardless of number of previous monovalent doses received). Patients who received only monovalent doses were included if they received any combination of 1–4 doses (or 1–5 doses if immunocompromised) monovalent mRNA (Moderna or Pfizer-BioNTech), Janssen (Johnson & Johnson), or Novavax vaccine doses; recipients of a single monovalent mRNA dose or a single Novavax dose were excluded. In addition, patients were excluded if any vaccine dose was received <7 days before the index date, if a bivalent dose was received ≥180 days before the index date, or if >1 bivalent dose was received.** Patients aged <50 years without documented immunocompromising conditions were excluded if they had received >3 monovalent doses. Patients were considered to have critical illness if they were admitted to an ICU, died, or both.^††^

Absolute VE was estimated using a test-negative case-control design comparing the odds of vaccination (either bivalent booster or monovalent doses only versus being unvaccinated) among case- and control patients. Relative VE was calculated by comparing those who received a bivalent booster with those who received monovalent doses only. A combined model was generated and included patients who had only received monovalent vaccination ≥7 days before their index date, or a bivalent mRNA booster dose at 7–59, 60–119, or 120–179 days before their index date, compared with an unvaccinated reference group. Odds ratios and 95% CIs were estimated using multivariable logistic regression controlling for age, race and ethnicity, sex, calendar day (days since January 1, 2021), and geographic region. Age and calendar day were modeled as natural cubic splines. VE was modeled separately for persons with and without immunocompromising conditions, by age group (18–64 and ≥65 years), and for each outcome (hospitalization and critical illness).^§§^ Analyses were conducted using R (version 4.2.2; The R Foundation). This study was conducted consistent with applicable federal law and CDC policy and was reviewed and approved by Institutional Review Boards at participating sites or under a reliance agreement with the Institutional Review Board of Westat, Inc.^¶¶^

Among 66,141 hospitalized patients without immunocompromising conditions who met inclusion criteria, 6,907 (10.4%) were case-patients and 59,234 (89.6%) were control patients (Table 1). Median age of case- and control patients was 76 years and 71 years, respectively. Among case- and control patients, 25.9% and 23.2% were unvaccinated, respectively; a bivalent vaccine dose had been received by 16.3% of case-patients and 20.6% of control patients. VE against COVID-19–associated hospitalization was similar across age groups, but waned over time, from 62% during the first 7–59 days after the bivalent dose to 24% by 120–179 days among adults aged ≥18 years (Table 2). Among those who received monovalent doses only, VE was 21% a median 376 days after the last dose. VE against critical illness was 69% during the 7–59 days after receipt of a bivalent dose and was more sustained (50% at 120–179 days after bivalent vaccination) than VE against hospitalization.

**TABLE 1:** Characteristics of hospitalizations among immunocompetent adults aged ≥18 years with COVID-19–like illness,* by COVID-19 vaccination status and SARS-CoV-2 test result — seven states,^†^ September 2022–April 2023

Characteristic	Overall, no. (column %)	SARS-CoV-2 test result status, no. (row %)		SMD^§^	Vaccination status,^¶^ no. (row %)					SMD^§^
		Case-patients (positive)	Control patients (negative)		Unvaccinated	Primary series with or without MV booster, ≥7 days earlier**	BV mRNA dose, 7–59 days earlier	BV mRNA dose, 60–119 days earlier	BV mRNA dose, 120–179 days earlier	
**All hospitalizations (row %)**	**66,141 (100.0)**	**6,907 (10.4)**	**59,234 (89.6)**	**NA**	**15,514 (23.5)**	**37,269 (56.3)**	**4,857 (7.3)**	**5,191 (7.8)**	**3,310 (5.0)**	**NA**
**Variant-predominant period^††^**										
BA.4/BA.5–related	**47,065 (71.2)**	5,219 (11.1)	41,846 (88.9)	0.11	11,240 (23.9)	27,594 (58.6)	4,439 (9.4)	3,525 (7.5)	267 (0.6)	1.09
XBB-related	**19,076 (28.8)**	1,688 (8.8)	17,388 (91.2)		4,274 (22.4)	9,675 (50.7)	418 (2.2)	1,666 (8.7)	3,043 (16.0)	
**Site**										
HealthPartners	**5,354 (8.1)**	646 (12.1)	4,708 (87.9)	0.11	969 (18.1)	2,716 (50.7)	678 (12.7)	643 (12.0)	348 (6.5)	2.72
Intermountain Healthcare	**7,572 (11.4)**	933 (12.3)	6,639 (87.7)		2,041 (27.0)	3,994 (52.7)	538 (7.1)	595 (7.9)	404 (5.3)	
KPCHR	**4,974 (7.5)**	414 (8.3)	4,560 (91.7)		1,232 (24.8)	2,565 (51.6)	458 (9.2)	465 (9.3)	254 (5.1)	
KPNC	**23,294 (35.2)**	2,271 (9.7)	21,023 (90.3)		2,147 (9.2)	15,152 (65.0)	2,017 (8.7)	2,338 (10.0)	1,640 (7.0)	
Regenstrief Institute	**24,947 (37.7)**	2,643 (10.6)	22,304 (89.4)		9,125 (36.6)	12,842 (51.5)	1,166 (4.7)	1,150 (4.6)	664 (2.7)	
**Age group, yrs**										
18–49	**9,656 (14.6)**	552 (5.7)	9,104 (94.3)	0.35	4,047 (41.9)	4,880 (50.5)	288 (3.0)	283 (2.9)	158 (1.6)	2.25
50–64	**13,200 (20.0)**	995 (7.5)	12,205 (92.5)		3,986 (30.2)	7,488 (56.7)	671 (5.1)	652 (4.9)	403 (3.1)	
65–74	**15,002 (22.7)**	1,496 (10.0)	13,506 (90.0)		3,206 (21.4)	8,531 (56.9)	1,240 (8.3)	1,242 (8.3)	783 (5.2)	
75–84	**16,791 (25.4)**	2,155 (12.8)	14,636 (87.2)		2,702 (16.1)	9,671 (57.6)	1,582 (9.4)	1,726 (10.3)	1,110 (6.6)	
≥85	**11,492 (17.4)**	1,709 (14.9)	9,783 (85.1)		1,573 (13.7)	6,699 (58.3)	1,076 (9.4)	1,288 (11.2)	856 (7.4)	
**Sex**										
Men	**30,327 (45.9)**	3,412 (11.3)	26,915 (88.7)	0.08	7,503 (24.7)	16,802 (55.4)	2,163 (7.1)	2,333 (7.7)	1,526 (5.0)	0.23
Women	**35,814 (54.1)**	3,495 (9.8)	32,319 (90.2)		8,011 (22.4)	20,467 (57.1)	2,694 (7.5)	2,858 (8.0)	1,784 (5.0)	
**Race and ethnicity**										
Black or African American, non-Hispanic	**5,953 (9.0)**	481 (8.1)	5,473 (91.9)	0.11	1,923 (32.3)	3,317 (55.7)	263 (4.4)	275 (4.6)	175 (2.9)	1.17
White, non-Hispanic	**45,101 (68.2)**	4,976 (11.0)	40,125 (89.0)		10,399 (23.1)	24,751 (54.9)	3,593 (8.0)	3,892 (8.6)	2,466 (5.5)	
Hispanic or Latino	**5,622 (8.5)**	517 (9.2)	5,105 (90.8)		944 (16.8)	3,736 (66.5)	364 (6.5)	350 (6.2)	228 (4.1)	
Other,^§§^ non-Hispanic	**6,054 (9.2)**	566 (9.3)	5,488 (90.7)		926 (15.3)	3,716 (61.4)	496 (8.2)	553 (9.1)	363 (6.0)	
Unknown	**3,411 (5.2)**	367 (10.8)	3,044 (89.2)		1,322 (38.8)	1,749 (51.3)	141 (4.1)	121 (3.5)	78 (2.3)	
**SARS-CoV-2 test result status**										
Case-patients (positive)	**6,907 (10.4)**	6,907 (100.0)	0 (—)	NA	1,791 (25.9)	3,988 (57.7)	327 (4.7)	486 (7.0)	315 (4.6)	NA
Control patients (negative)	**59,234 (89.6)**	0 (—)	59,234 (100.0)		13,723 (23.2)	33,281 (56.2)	4,530 (7.6)	4,705 (7.9)	2,995 (5.1)	
**No. of MV doses received**										
0	**15,599 (23.6)**	1,798 (11.5)	13,801 (88.5)	NA	15,514 (99.5)	0 (—)	36 (0.2)	30 (0.2)	19 (0.1)	NA
1	**1,402 (2.1)**	116 (8.3)	1,286 (91.7)		0 (—)	1,259 (89.8)	53 (3.8)	54 (3.9)	36 (2.6)	
2	**12,948 (19.6)**	1,340 (10.3)	11,608 (89.7)		0 (—)	11,936 (92.2)	404 (3.1)	407 (3.1)	201 (1.6)	
3	**21,860 (33.1)**	2,199 (10.1)	19,661 (89.9)		0 (—)	16,684 (76.3)	1,959 (9.0)	1,972 (9.0)	1,245 (5.7)	
4	**14,332 (21.7)**	1,454 (10.1)	12,878 (89.9)		0 (—)	7,390 (51.6)	2,405 (16.8)	2,728 (19.0)	1,809 (12.6)	
**MV product received, by manufacturer^¶¶^**										
Pfizer-BioNTech	**29,721 (58.7)**	2,950 (9.9)	26,771 (90.1)	NA	NA	21,435 (72.1)	3,018 (10.1)	3,203 (10.8)	2,065 (6.9)	NA
Moderna	**23,048 (45.5)**	2,384 (10.3)	20,664 (89.7)		NA	16,887 (73.3)	2,242 (9.7)	2,409 (10.4)	1,510 (6.6)	
Janssen (Johnson & Johnson)	**3,230 (6.4)**	286 (8.9)	2,944 (91.1)		NA	2,770 (85.8)	176 (5.4)	177 (5.5)	107 (3.3)	
Novavax	**1 (0)**	0 (—)	1 (100.0)		NA	1 (100.0)	0 (—)	0 (—)	0 (—)	
**COVID-19 vaccination status**										
Unvaccinated	**15,514 (23.5)**	1,791 (11.5)	13,723 (88.5)	0.14	15,514 (100.0)	0 (—)	0 (—)	0 (—)	0 (—)	NA
Primary series with or without MV booster	**37,269 (56.3)**	3,988 (10.7)	33,281 (89.3)		0 (—)	37,269 (100.0)	0 (—)	0 (—)	0 (—)	
mRNA BV dose, 7–59 days earlier	**4,857 (7.3)**	327 (6.7)	4,530 (93.3)		0 (—)	0 (—)	4,857 (100.0)	0 (—)	0 (—)	
mRNA BV dose, 60–119 days earlier	**5,191 (7.8)**	486 (9.4)	4,705 (90.6)		0 (—)	0 (—)	0 (—)	5,191 (100.0)	0 (—)	
mRNA BV dose, 120–179 days earlier	**3,310 (5.0)**	315 (9.5)	2,995 (90.5)		0 (—)	0 (—)	0 (—)	0 (—)	3,310 (100.0)	
**Most recent dose product manufacturer**										
Pfizer-BioNTech	**29,892 (59.0)**	2,968 (9.9)	26,924 (90.1)	0.04	0 (—)	20,271 (67.8)	3,441 (11.5)	3,732 (12.5)	2,448 (8.2)	NA
Moderna	**19,084 (37.7)**	2,004 (10.5)	17,080 (89.5)		0 (—)	15,347 (80.4)	1,416 (7.4)	1,459 (7.6)	862 (4.5)	
Janssen (Johnson & Johnson)	**1,650 (3.3)**	144 (8.7)	1,506 (91.3)		0 (—)	1,650 (100.0)	0 (—)	0 (—)	0 (—)	
Novavax	**1 (0)**	0 (—)	1 (100.0)		0 (—)	1 (100.0)	0 (—)	0 (—)	0 (—)	
**One or more chronic respiratory condition**										
Yes	**39,469 (59.7)**	4,286 (10.9)	35,183 (89.1)	0.05	8,602 (21.8)	22,383 (56.7)	3,067 (7.8)	3,314 (8.4)	2,103 (5.3)	0.46
No	**26,672 (40.3)**	2,621 (9.8)	24,051 (90.2)		6,912 (25.9)	14,886 (55.8)	1,790 (6.7)	1,877 (7.0)	1,207 (4.5)	
**One or more chronic nonrespiratory condition**										
Yes	**54,754 (82.8)**	5,843 (10.7)	48,911 (89.3)	0.05	11,322 (20.7)	31,413 (57.4)	4,344 (7.9)	4,679 (8.5)	2,996 (5.5)	1.28
No	**11,387 (17.2)**	1,064 (9.3)	10,323 (90.7)		4,192 (36.8)	5,856 (51.4)	513 (4.5)	512 (4.5)	314 (2.7)	
**ICU admission**										
Yes	**12,197 (18.4)**	1,023 (8.4)	11,174 (91.6)	0.11	3,146 (25.8)	6,763 (55.4)	848 (7.0)	886 (7.3)	554 (4.5)	0.22
No	**53,944 (81.6)**	5,884 (10.9)	48,060 (89.1)		12,368 (22.9)	30,506 (56.6)	4,009 (7.4)	4,305 (8.0)	2,756 (5.1)	
**Receipt of invasive mechanical ventilation**										
Yes	**3,293 (5.0)**	250 (7.6)	3,043 (92.4)	0.08	804 (24.4)	1,857 (56.4)	229 (7.0)	254 (7.7)	149 (4.5)	1.89
No	**44,995 (68.0)**	4,814 (10.7)	40,181 (89.3)		7,984 (17.7)	26,340 (58.5)	3,850 (8.6)	4,151 (9.2)	2,670 (5.9)	
Unknown	**17,853 (27.0)**	1,843 (10.3)	16,010 (89.7)		6,726 (37.7)	9,072 (50.8)	778 (4.4)	786 (4.4)	491 (2.8)	
In-hospital death***										
Yes	**2,735 (4.1)**	331 (12.1)	2,404 (87.9)	0.04	727 (26.6)	1,433 (52.4)	183 (6.7)	246 (9.0)	146 (5.3)	0.09
No	**63,406 (95.9)**	6,576 (10.4)	56,830 (89.6)		14,787 (23.3)	35,836 (56.5)	4,674 (7.4)	4,945 (7.8)	3,164 (5.0)	

**Abbreviations:** BV = bivalent; ICU = intensive care unit; KPCHR = Kaiser Permanente Center for Health Research; KPNC = Kaiser Permanente Northern California; MV = monovalent; NA = not applicable; SMD = standardized mean or proportion difference.

* Hospitalizations with a discharge code consistent with COVID-19–like illness were included. COVID-19–like illness diagnoses included acute respiratory illness, respiratory signs or symptoms or febrile signs or symptoms using diagnosis codes from the *International Classification of Diseases, Tenth Revision*. Clinician-ordered molecular assays (e.g., real-time reverse transcription–polymerase chain reaction) for SARS-CoV-2 occurring ≤14 days before to <72 hours after the date of admission were included.

^†^ California (September 13, 2022–April 21, 2023), Indiana (September 13, 2022–April 12, 2023), Minnesota and Wisconsin (September 13, 2022–April 21, 2023), Oregon and Washington (September 13, 2022–April 14, 2023), and Utah (September 13, 2022–April 21, 2023).

^§^ An absolute SMD >0.20 indicates a nonnegligible difference in variable distributions between hospitalizations for vaccinated versus unvaccinated patients, or for patients with a positive SARS-CoV-2 test result versus patients with a negative SARS-CoV-2 test result. For mRNA COVID-19 vaccination status, a single SMD was calculated by averaging the absolute SMDs obtained from pairwise comparisons of each vaccinated category versus unvaccinated. Specifically, it was calculated as the average of the absolute value of the SMDs for 1) vaccinated with only MV doses, ≥7 days earlier versus unvaccinated, 2) vaccinated with an mRNA BV dose, 7–59 days earlier versus unvaccinated, 3) vaccinated with an mRNA BV dose, 60–119 days earlier versus unvaccinated, and 4) vaccinated with an mRNA BV dose, 120–179 days earlier versus unvaccinated.

^¶^ Vaccination was defined as having received the last MV or BV dose within the specified range of days before the index date, which was the date of respiratory specimen collection associated with the most recent positive or negative SARS-CoV-2 test result before the admission date, or the admission date if testing only occurred after the admission.

** Includes persons who received a single dose of Janssen (Johnson & Johnson) vaccine. Persons who received a single dose of Pfizer-BioNTech, Moderna, or Novavax vaccine were excluded from the analysis.

^††^ Variant predominance was defined as the period when a variant accounted for ≥50% of all sequenced specimens in the U.S. Department of Health and Human Services region where the site is located. XBB-related sublineages predominated at Intermountain Healthcare beginning January 28, 2023; at HealthPartners, KPNC, and Regenstrief Institute beginning February 4, 2023; and at KPCHR beginning February 11, 2023.

^§§^ Other race includes American Indian or Alaska Native, Asian, Native Hawaiian or other Pacific Islander, other races not listed, and multiple races. Because of small numbers, these categories were combined.

^¶¶^ Because persons might have received vaccine from more than one manufacturer, columns might sum to >100%.

*** In-hospital death was identified at each individual site and was defined as a death while hospitalized or ≤28 days after admission.

**TABLE 2:** COVID-19 vaccine effectiveness* against laboratory-confirmed COVID-19–associated hospitalizations and critical illness^†^ among adults aged ≥18 years, by age group and immunocompromise status — seven states,^§^ September 2022–April 2023

Clinical status/Age group, yrs/Vaccine type and doses received, interval since receipt of BV dose	Without documented immunocompromising conditions				With documented immunocompromising conditions			
	Total	Positive SARS-CoV-2 test result, no. (%)	Median interval since last dose, days (IQR)	VE, % (95% CI)	Total	Positive SARS-CoV-2 test result, no. (%)	Median interval since last dose, days (IQR)	VE, % (95% CI)
**Hospitalization**								
**≥18**								
Unvaccinated (Ref)	**15,514**	1,791 (11.5)	NA	Ref	**3,109**	314 (10.1)	NA	Ref
MV only	**37,269**	3,988 (10.7)	376 (270 to 505)	21 (16 to 26)	**11,140**	1,134 (10.2)	355 (237 to 474)	3 (−12 to 16)
BV, 7–59 days earlier	**4,857**	327 (6.7)	34 (21 to 47)	62 (57 to 67)	**1,612**	143 (8.9)	33 (19 to 46)	28 (10 to 42)
BV, 60–119 days earlier	**5,191**	486 (9.4)	87 (73 to 103)	47 (41 to 53)	**1,829**	140 (7.6)	88 (74 to 104)	41 (26 to 53)
BV, 120–179 days earlier	**3,310**	315 (9.5)	144 (132 to 159)	24 (12 to 33)	**1,244**	103 (8.3)	144 (131 to 159)	13 (−13 to 33)
**18–64**								
Unvaccinated (Ref)	**8,033**	591 (7.4)	NA	Ref	**NA**	NA	NA	NA
MV only	**12,368**	821 (6.6)	403 (306 to 534)	17 (7 to 26)	**NA**	NA	NA	NA
BV, 7–59 days earlier	**959**	38 (4.0)	33 (21 to 45)	61 (44 to 72)	**NA**	NA	NA	NA
BV, 60–119 days earlier	**935**	66 (7.1)	86 (72 to 101)	25 (1 to 43)	**NA**	NA	NA	NA
BV, 120–179 days earlier	**561**	31 (5.5)	143 (131 to 158)	16 (−24 to 43)^¶^	**NA**	NA	NA	NA
**≥65**								
Unvaccinated (Ref)	**7,481**	1,200 (16.0)	NA	Ref	**NA**	NA	NA	NA
MV only	**24,901**	3,167 (12.7)	362 (245 to 484)	24 (18 to 29)	**NA**	NA	NA	NA
BV, 7–59 days earlier	**3,898**	289 (7.4)	35 (21 to 48)	64 (58 to 68)	**NA**	NA	NA	NA
BV, 60–119 days earlier	**4,256**	420 (9.9)	87 (73 to 103)	51 (45 to 57)	**NA**	NA	NA	NA
BV, 120–179 days earlier	**2,749**	284 (10.3)	145 (132 to 159)	27 (15 to 37)	**NA**	NA	NA	NA
Critical illness^**^								
**≥18**								
Unvaccinated (Ref)	**14,090**	367 (2.6)	NA	Ref	**2,881**	86 (3.0)	NA	Ref
MV only	**33,925**	644 (1.9)	375 (269 to 505)	31 (21 to 40)	**10,263**	257 (2.5)	354 (235 to 474)	16 (−10 to 36)
BV, 7–59 days earlier	**4,579**	49 (1.1)	34 (21 to 47)	69 (57 to 77)	**1,501**	32 (2.1)	33 (19 to 46)	40 (7 to 61)^¶^
BV, 60–119 days earlier	**4,790**	85 (1.8)	86 (73 to 103)	46 (30 to 58)	**1,725**	36 (2.1)	88 (74 to 104)	43 (14 to 63)
BV, 120–179 days earlier	**3,028**	33 (1.1)	144 (132 to 159)	50 (26 to 66)	**1,155**	14 (1.2)	144 (131 to 159)	53 (13 to 75)^¶^

**Abbreviations:** BV = bivalent; MV = monovalent; NA = not applicable; Ref = referent group; VE = vaccine effectiveness.

* VE was calculated as (1 − odds ratio) x 100%, estimated using a test-negative case-control design, adjusted for age, sex, race and ethnicity, geographic region, and calendar time (days since January 1, 2021).

^†^ Patients were considered to have critical illness if they were admitted to an intensive care unit or died. Death was identified at each individual site and was defined as a death while hospitalized or ≤28 days after admission.

^§^ California (September 13, 2022–April 21, 2023), Indiana (September 13, 2022–April 12, 2023), Minnesota and Wisconsin (September 13, 2022–April 21, 2023), Oregon and Washington (September 13, 2022–April 14, 2023), and Utah (September 13, 2022–April 21, 2023).

^¶^ These estimates are imprecise, which might be because of a relatively small number of persons in each level of vaccination or case status. This imprecision indicates the actual VE could be substantially different from the point estimate shown, and estimates should therefore be interpreted with caution. Additional data accrual could increase precision and allow appropriate interpretation.

** For VE against critical illness, case-patients were persons admitted to an intensive care unit or who experienced death associated with COVID-19, and control patients were persons hospitalized without COVID-19.

Among 18,934 hospitalized patients with immunocompromising conditions who met inclusion criteria, 1,834 (9.7%) were case-patients and 17,100 (90.3%) were control patients (Table 3); these persons represented 22.3% of the overall hospitalized population who met inclusion criteria. Median age of case- and control patients was 73 years and 70 years, respectively. Within this group, 17.1% of case-patients and 16.3% of control patients were unvaccinated; 21.0% of case-patients and 25.1% of control patients had received a bivalent dose. Among patients aged ≥18 years with immunocompromising conditions, VE against COVID-19–associated hospitalization was 28% during the first 7–59 days after receipt of the bivalent dose and declined to 13% by 120–179 days. VE for those who received monovalent doses only was 3% (median 355 days after the last dose). Estimates of relative and absolute VE were similar (Supplementary Table, https://stacks.cdc.gov/view/cdc/128421).

**TABLE 3:** Characteristics of hospitalizations among immunocompromised adults aged ≥18 years with COVID-19–like illness,* by COVID-19 vaccination status and SARS-CoV-2 test result — seven states,^†^ September 2022–April 2023

Characteristic	Overall, no. (column %)	SARS-CoV-2 test result status, no. (row %)		SMD^§^	Vaccination status,^¶^ no. (row %)					SMD^§^
		Case-patients (positive)	Control patients (negative)		Unvaccinated	Primary series with or without MV booster, ≥7 days earlier**	BV mRNA dose, 7–59 days earlier	BV mRNA dose, 60–119 days earlier	BV mRNA dose, 120–179 days earlier	
**All hospitalizations (row %)**	**18,934 (100.0)**	**1,834 (9.7)**	**17,100 (90.3)**	**NA**	**3,109 (16.4)**	**11,140 (58.8)**	**1,612 (8.5)**	**1,829 (9.7)**	**1,244 (6.6)**	**NA**
**Variant-predominant period^††^**										
BA.4/BA.5–related	**13,310 (70.3)**	1,347 (10.1)	11,963 (89.9)	0.08	2,261 (17.0)	8,282 (62.2)	1,480 (11.1)	1,196 (9.0)	91 (0.7)	1.17
XBB-related	**5,624 (29.7)**	487 (8.7)	5,137 (91.3)		848 (15.1)	2,858 (50.8)	132 (2.3)	633 (11.3)	1,153 (20.5)	
**Site**										
HealthPartners	**1,896 (10.0)**	217 (11.4)	1,679 (88.6)	0.13	288 (15.2)	1,014 (53.5)	244 (12.9)	218 (11.5)	132 (7.0)	2.7
Intermountain Healthcare	**2,092 (11.0)**	244 (11.7)	1,848 (88.3)		446 (21.3)	1,171 (56.0)	154 (7.4)	188 (9.0)	133 (6.4)	
KPCHR	**1,633 (8.6)**	132 (8.1)	1,501 (91.9)		346 (21.2)	853 (52.2)	167 (10.2)	161 (9.9)	106 (6.5)	
KPNC	**8,957 (47.3)**	795 (8.9)	8,162 (91.1)		652 (7.3)	5,726 (63.9)	823 (9.2)	1,016 (11.3)	740 (8.3)	
Regenstrief Institute	**4,356 (23.0)**	446 (10.2)	3,910 (89.8)		1,377 (31.6)	2,376 (54.5)	224 (5.1)	246 (5.6)	133 (3.1)	
**Age group, yrs**										
18–49	**2,080 (11.0)**	140 (6.7)	1,940 (93.3)	0.21	677 (32.5)	1,174 (56.4)	95 (4.6)	77 (3.7)	57 (2.7)	2.03
50–64	**4,220 (22.3)**	351 (8.3)	3,869 (91.7)		892 (21.1)	2,610 (61.8)	259 (6.1)	290 (6.9)	169 (4.0)	
65–74	**5,567 (29.4)**	508 (9.1)	5,059 (90.9)		808 (14.5)	3,255 (58.5)	492 (8.8)	585 (10.5)	427 (7.7)	
75–84	**4,807 (25.4)**	550 (11.4)	4,257 (88.6)		526 (10.9)	2,795 (58.1)	501 (10.4)	582 (12.1)	403 (8.4)	
≥85	**2,260 (11.9)**	285 (12.6)	1,975 (87.4)		206 (9.1)	1,306 (57.8)	265 (11.7)	295 (13.1)	188 (8.3)	
**Sex**										
Men	**9,187 (48.5)**	930 (10.1)	8,257 (89.9)	0.05	1,512 (16.5)	5,334 (58.1)	773 (8.4)	921 (10.0)	647 (7.0)	0.08
Women	**9,747 (51.5)**	904 (9.3)	8,843 (90.7)		1,597 (16.4)	5,806 (59.6)	839 (8.6)	908 (9.3)	597 (6.1)	
**Race and ethnicity**										
Black or African American, non-Hispanic	**1,609 (8.5)**	155 (9.6)	1,454 (90.4)	0.06	292 (18.1)	1,008 (62.6)	132 (8.2)	108 (6.7)	69 (4.3)	1.09
White, non-Hispanic	**12,656 (66.8)**	1,250 (9.9)	11,406 (90.1)		2,156 (17.0)	7,142 (56.4)	1,148 (9.1)	1,324 (10.5)	886 (7.0)	
Hispanic or Latino	**2,027 (10.7)**	192 (9.5)	1,835 (90.5)		255 (12.6)	1,366 (67.4)	138 (6.8)	168 (8.3)	100 (4.9)	
Other,^§§^ non-Hispanic	**2,064 (10.9)**	172 (8.3)	1,892 (91.7)		191 (9.3)	1,325 (64.2)	173 (8.4)	199 (9.6)	176 (8.5)	
Unknown	**578 (3.1)**	65 (11.2)	513 (88.8)		215 (37.2)	299 (51.7)	21 (3.6)	30 (5.2)	13 (2.2)	
**SARS-CoV-2 test result status**										
Case-patients (positive)	**1,834 (9.7)**	1,834 (100.0)	0 (—)	NA	314 (17.1)	1,134 (61.8)	143 (7.8)	140 (7.6)	103 (5.6)	NA
Control patients (negative)	**17,100 (90.3)**	0 (—)	17,100 (100.0)		2,795 (16.3)	10,006 (58.5)	1,469 (8.6)	1,689 (9.9)	1,141 (6.7)	
**No. of MV doses received**										
0	**3,133 (16.5)**	315 (10.1)	2,818 (89.9)	NA	3,109 (99.2)	0 (—)	6 (0.2)	15 (0.5)	3 (0.1)	NA
1	**332 (1.8)**	34 (10.2)	298 (89.8)		0 (—)	296 (89.2)	15 (4.5)	15 (4.5)	6 (1.8)	
2	**3,207 (16.9)**	289 (9.0)	2,918 (91.0)		0 (—)	2,951 (92.0)	87 (2.7)	107 (3.3)	62 (1.9)	
3	**6,376 (33.7)**	649 (10.2)	5,727 (89.8)		0 (—)	4,854 (76.1)	579 (9.1)	580 (9.1)	363 (5.7)	
4	**5,689 (30.0)**	523 (9.2)	5,166 (90.8)		0 (—)	2,903 (51.0)	902 (15.9)	1,093 (19.2)	791 (13.9)	
5	**197 (1.0)**	24 (12.2)	173 (87.8)		0 (—)	136 (69.0)	23 (11.7)	19 (9.6)	19 (9.6)	
**MV product received, by manufacturer^¶¶^**										
Pfizer-BioNTech	**9,634 (60.9)**	937 (9.7)	8,697 (90.3)	NA	NA	6,644 (69.0)	1,021 (10.6)	1,167 (12.1)	802 (8.3)	NA
Moderna	**6,923 (43.7)**	659 (9.5)	6,264 (90.5)		NA	4,879 (70.5)	715 (10.3)	788 (11.4)	541 (7.8)	
Janssen (Johnson & Johnson)	**982 (6.2)**	89 (9.1)	893 (90.9)		NA	821 (83.6)	55 (5.6)	59 (6.0)	47 (4.8)	
Novavax	**3 (0)**	1 (33.3)	2 (66.7)		NA	3 (100.0)	0 (—)	0 (—)	0 (—)	
**COVID-19 vaccination status**										
Unvaccinated	**3,109 (16.4)**	314 (10.1)	2,795 (89.9)	NA	3,109 (100.0)	0 (—)	0 (—)	0 (—)	0 (—)	NA
Primary series with or without MV booster dose(s)	**11,140 (58.8)**	1,134 (10.2)	10,006 (89.8)		0 (—)	11,140 (100.0)	0 (—)	0 (—)	0 (—)	
mRNA BV dose, 7–59 days earlier	**1,612 (8.5)**	143 (8.9)	1,469 (91.1)		0 (—)	0 (—)	1,612 (100.0)	0 (—)	0 (—)	
mRNA BV dose, 60–119 days earlier	**1,829 (9.7)**	140 (7.7)	1,689 (92.3)		0 (—)	0 (—)	0 (—)	1,829 (100.0)	0 (—)	
mRNA BV dose, 120–179 days earlier	**1,244 (6.6)**	103 (8.3)	1,141 (91.7)		0 (—)	0 (—)	0 (—)	0 (—)	1,244 (100.0)	
**Most recent dose product manufacturer**										
Pfizer-BioNTech	**9,757 (61.7)**	936 (9.6)	8,821 (90.4)	0.03	0 (—)	6,317 (64.7)	1,179 (12.1)	1,335 (13.7)	926 (9.5)	NA
Moderna	**5,646 (35.7)**	546 (9.7)	5,100 (90.3)		0 (—)	4,401 (77.9)	433 (7.7)	494 (8.7)	318 (5.6)	
Janssen (Johnson & Johnson)	**419 (2.6)**	37 (8.8)	382 (91.2)		0 (—)	419 (100.0)	0 (—)	0 (—)	0 (—)	
Novavax	**3 (0)**	1 (33.3)	2 (66.7)		0 (—)	3 (100.0)	0 (—)	0 (—)	0 (—)	
**One or more chronic respiratory condition**										
Yes	**12,146 (64.1)**	1,259 (10.4)	10,887 (89.6)	0.11	1,966 (16.2)	7,102 (58.5)	1,056 (8.7)	1,209 (10.0)	813 (6.7)	0.13
No	**6,788 (35.9)**	575 (8.5)	6,213 (91.5)		1,143 (16.8)	4,038 (59.5)	556 (8.2)	620 (9.1)	431 (6.3)	
**One or more chronic nonrespiratory condition**										
Yes	**17,973 (94.9)**	1,753 (9.8)	16,220 (90.2)	0.11	2,855 (15.9)	10,600 (59.0)	1,554 (8.6)	1,771 (9.9)	1,193 (6.6)	0.13
No	**961 (5.1)**	81 (8.4)	880 (91.6)		254 (26.4)	540 (56.2)	58 (6.0)	58 (6.0)	51 (5.3)	
**Solid malignancy**										
Yes	**8,202 (43.3)**	639 (7.8)	7,563 (92.2)	0.19	1,240 (15.1)	4,871 (59.4)	712 (8.7)	822 (10.0)	557 (6.8)	0.3
No	**10,732 (56.7)**	1,195 (11.1)	9,537 (88.9)		1,869 (17.4)	6,269 (58.4)	900 (8.4)	1,007 (9.4)	687 (6.4)	
**Hematologic malignancy**										
Yes	**2,775 (14.7)**	324 (11.7)	2,451 (88.3)	0.09	409 (14.7)	1,639 (59.1)	250 (9.0)	294 (10.6)	183 (6.6)	0.2
No	**16,159 (85.3)**	1,510 (9.3)	14,649 (90.7)		2,700 (16.7)	9,501 (58.8)	1,362 (8.4)	1,535 (9.5)	1,061 (6.6)	
**Rheumatologic or inflammatory disorder**										
Yes	**4,752 (25.1)**	550 (11.6)	4,202 (88.4)	0.12	746 (15.7)	2,752 (57.9)	458 (9.6)	488 (10.3)	308 (6.5)	0.19
No	**14,182 (74.9)**	1,284 (9.1)	12,898 (90.9)		2,363 (16.7)	8,388 (59.1)	1,154 (8.1)	1,341 (9.5)	936 (6.6)	
**Other intrinsic immune condition or immunodeficiency**										
Yes	**6,056 (32.0)**	647 (10.7)	5,409 (89.3)	0.08	1,105 (18.2)	3,593 (59.3)	456 (7.5)	534 (8.8)	368 (6.1)	0.4
No	**12,878 (68.0)**	1,187 (9.2)	11,691 (90.8)		2,004 (15.6)	7,547 (58.6)	1,156 (9.0)	1,295 (10.1)	876 (6.8)	
**Organ or stem cell transplant**										
Yes	**1,298 (6.9)**	172 (13.3)	1,126 (86.7)	0.1	162 (12.5)	783 (60.3)	122 (9.4)	133 (10.2)	98 (7.6)	0.3
No	**17,636 (93.1)**	1,662 (9.4)	15,974 (90.6)		2,947 (16.7)	10,357 (58.7)	1,490 (8.4)	1,696 (9.6)	1,146 (6.5)	
**HIV/AIDS**										
Yes	**350 (1.8)**	30 (8.6)	320 (91.4)	0.02	79 (22.6)	191 (54.6)	32 (9.1)	26 (7.4)	22 (6.3)	0.19
No	**18,584 (98.2)**	1,804 (9.7)	16,780 (90.3)		3,030 (16.3)	10,949 (58.9)	1,580 (8.5)	1,803 (9.7)	1,222 (6.6)	
**ICU admission**										
Yes	**4,094 (21.6)**	335 (8.2)	3,759 (91.8)	0.09	747 (18.2)	2,411 (58.9)	323 (7.9)	367 (9.0)	246 (6.0)	0.29
No	**14,840 (78.4)**	1,499 (10.1)	13,341 (89.9)		2,362 (15.9)	8,729 (58.8)	1,289 (8.7)	1,462 (9.9)	998 (6.7)	
**Receipt of invasive mechanical ventilation**										
Yes	**1,403 (7.4)**	131 (9.3)	1,272 (90.7)	0.02	274 (19.5)	839 (59.8)	99 (7.1)	114 (8.1)	77 (5.5)	1.47
No	**15,220 (80.4)**	1,467 (9.6)	13,753 (90.4)		2,104 (13.8)	9,038 (59.4)	1,402 (9.2)	1,589 (10.4)	1,087 (7.1)	
Unknown	**2,311 (12.2)**	236 (10.2)	2,075 (89.8)		731 (31.6)	1,263 (54.7)	111 (4.8)	126 (5.5)	80 (3.5)	
In-hospital death***										
Yes	**1,638 (8.7)**	169 (10.3)	1,469 (89.7)	0.02	336 (20.5)	946 (57.8)	102 (6.2)	155 (9.5)	99 (6.0)	0.35
No	**17,296 (91.3)**	1,665 (9.6)	15,631 (90.4)		2,773 (16.0)	10,194 (58.9)	1,510 (8.7)	1,674 (9.7)	1,145 (6.6)	

**Abbreviations:** BV = bivalent; ICU = intensive care unit; KPCHR = Kaiser Permanente Center for Health Research; KPNC = Kaiser Permanente Northern California; MV = monovalent; NA = not applicable; SMD = standardized mean or proportion difference.

* Hospitalizations with a discharge code consistent with COVID-19–like illness were included. COVID-19–like illness diagnoses included acute respiratory illness, respiratory signs or symptoms or febrile signs or symptoms using diagnosis codes from the *International Classification of Diseases, Tenth Revision*. Clinician-ordered molecular assays (e.g., real-time reverse transcription–polymerase chain reaction) for SARS-CoV-2 occurring ≤14 days before to <72 hours after the date of admission were included.

^†^ California (September 13, 2022–April 21, 2023), Indiana (September 13, 2022–April 12, 2023), Minnesota and Wisconsin (September 13, 2022–April 21, 2023), Oregon and Washington (September 13, 2022–April 14, 2023), and Utah (September 13, 2022–April 21, 2023).

^§^ An absolute SMD >0.20 indicates a nonnegligible difference in variable distributions between hospitalizations for vaccinated versus unvaccinated patients or for patients with a positive SARS-CoV-2 test result versus patients with a negative SARS-CoV-2 test result. For mRNA COVID-19 vaccination status, a single SMD was calculated by averaging the absolute SMDs obtained from pairwise comparisons of each vaccinated category versus unvaccinated. Specifically, it was calculated as the average of the absolute value of the SMDs for 1) vaccinated with only MV doses, ≥7 days earlier versus unvaccinated, 2) vaccinated with an mRNA BV dose, 7–59 days earlier versus unvaccinated, 3) vaccinated with an mRNA BV dose, 60–119 days earlier versus unvaccinated, and 4) vaccinated with an mRNA BV dose, 120–179 days earlier versus unvaccinated.

^¶^ Vaccination was defined as having received the last MV or BV dose within the specified range of days before the index date, which was the date of respiratory specimen collection associated with the most recent positive or negative SARS-CoV-2 test result before the admission date or the admission date if testing only occurred after the admission.

** Includes persons who received a single dose of Janssen (Johnson & Johnson) vaccine. Persons who received a single dose of Pfizer-BioNTech, Moderna, or Novavax vaccine were excluded from the analysis.

^††^ Variant predominance was defined as the period when a variant accounted for ≥50% of all sequenced specimens in the U.S. Department of Health and Human Services region where the site is located. XBB-related sublineages predominated at Intermountain Healthcare beginning January 28, 2023; at HealthPartners, KPNC, and Regenstrief Institute beginning February 4, 2023; and at KPCHR beginning February 11, 2023.

^§§^ Other race includes American Indian or Alaska Native, Asian, Native Hawaiian or other Pacific Islander, other races not listed, and multiple races. Because of small numbers, these categories were combined.

^¶¶^ Because persons might have received vaccine from more than one manufacturer, columns might sum to >100%.

*** In-hospital death was identified at each individual site and was defined as a death while hospitalized or ≤28 days after admission.

## Discussion

In this multistate analysis of 85,075 hospitalizations of persons with COVID-19–like illness, bivalent doses were 62% effective among adults without immunocompromising conditions, and 28% effective in those with immunocompromising conditions in preventing COVID-19–associated hospitalization during the first 7–59 days after vaccination. Waning was evident in adults without immunocompromising conditions from 60–179 days (2–6 months) after vaccination. VE was more sustained against critical illness (50% at 120–179 days after vaccination) in adults without immunocompromising conditions, which suggests that bivalent vaccines provide durable protection against the most severe outcomes from COVID-19.

In this analysis, receipt of a bivalent dose boosted protection against COVID-19–associated hospitalization that had waned since receipt of previous monovalent doses; however, protection afforded by a bivalent dose against COVID-19–associated hospitalization in adults without immunocompromising conditions waned in a similar pattern to that seen after receipt of a monovalent dose during Omicron predominance, with high initial VE and a decrease over time since the last dose. Among adults without immunocompromising conditions, bivalent VE was similar against COVID-19–associated hospitalization and critical illness within the first 2 months after vaccination but appeared to be more durable against critical illness, consistent with previous CDC research showing durable protection by monovalent mRNA vaccines against critical illness, defined as invasive mechanical ventilation or death (*3*). Although VE point estimates were lower among persons with immunocompromising conditions compared with those without such conditions, waning was not evident in this group, possibly because of heterogeneity in immune response among those with immunocompromising conditions or because of limited statistical power to detect differences by time. Previous analyses have shown differences in VE by type of immunocompromising condition (*4*); however, this analysis did not have sufficient statistical power to differentiate VE by condition type.

 As of May 10, 2023, only one in five (20.5%) U.S. adults had received a bivalent booster dose.*** Among U.S. adults who previously received a monovalent vaccine but had yet to receive a bivalent mRNA booster, most received their last vaccine dose >1 year ago.^†††^ Results of this analysis indicate that these adults might have relatively little remaining protection against COVID-19–associated hospitalization compared with unvaccinated persons, although might have more remaining protection against critical illness.

On April 19, 2023, CDC amended recommendations to permit adults aged ≥65 years and those with immunocompromising conditions to receive ≥1 additional bivalent dose. In this analysis, waning VE patterns were the same in younger and older adults; however, rates of COVID-19–associated hospitalization and death remain substantially higher among older adults, which suggests that an additional dose might confer additional benefit. In addition, although this analysis did not demonstrate clear evidence of waning VE in immunocompromised adults, overall VE among immunocompromised adults was lower than among those without immunocompromising conditions. Like older adults, persons with immunocompromising conditions remain at higher risk for COVID-19 hospitalization and death and might benefit from additional bivalent doses, although this will require future evaluation.

The findings in this study are subject to at least six limitations. First, previous SARS-CoV-2 infection was not accounted for in this analysis. According to a national seroprevalence survey, a large proportion of the population has now experienced SARS-CoV-2 infection; infection-induced immunity decreases the risk for future medically attended COVID-19 illness and might affect observed VE.^§§§^ The findings of this analysis should therefore be interpreted in the context of this underlying immunity as the incremental benefit provided by COVID-19 vaccination. Second, although all case-patients included in the analysis had COVID-19–like illness and a positive SARS-CoV-2 test result at the time of the included hospitalization, some might have had relatively mild COVID-19 disease and been hospitalized because of reasons unrelated to COVID-19, which could lower measured VE. Third, although models adjusted for relevant confounders, such as age and calendar time, residual confounding is possible, including by behavioral differences, history of previous SARS-CoV-2 infection, and use of COVID-19 treatments such as nirmatrelvir-ritonavir (Paxlovid). Fourth, differences in sublineage-specific VE could not be compared because of limited statistical power. Fifth, this analysis did not compare product-specific bivalent booster VE estimates. Finally, because these data are from seven states, the patients in this analysis might not be representative of the entire U.S. population.

In this study of durability of bivalent VE, bivalent doses helped provide protection against COVID-19–associated hospitalization and critical disease. Although waning of protection was evident in some groups, VE was more sustained for critical illness, indicating the vaccines are continuing to help protect adults from the most severe COVID-19 outcomes. All adults should stay up to date with recommended COVID-19 vaccines.

**Summary**

**What is already known about this topic?**

Bivalent mRNA COVID-19 vaccines help provide protection against medically attended COVID-19–associated illness. However, the durability of this protection is uncertain.

**What is added by this report?**

Among adults aged ≥18 years without immunocompromising conditions, bivalent booster vaccine effectiveness (VE) against COVID-19–associated hospitalization declined from 62% at 7–59 days postvaccination to 24% at 120–179 days compared with VE among unvaccinated adults. Among immunocompromised adults, lower bivalent booster VE was observed. However, bivalent booster VE was sustained against critical COVID-19–associated outcomes, including intensive care unit admission or death.

**What are the implications for public health practice?**

Adults should stay up to date with recommended COVID-19 vaccines. Optional additional bivalent vaccine doses are available for older adults and persons with immunocompromising conditions.

## Footnotes

* Sites from the CDC-funded VISION Network that contributed data for this analysis were HealthPartners (Minnesota and Wisconsin), Intermountain Healthcare (Utah), Kaiser Permanente Northern California (California), Kaiser Permanente Center for Health Research (Oregon and Washington), and Regenstrief Institute (Indiana).

† Medical events with a discharge code consistent with COVID-19–like illness were included. COVID-19–like illness diagnoses were obtained from ICD-10 discharge codes. The specific codes used were COVID-19 pneumonia: J12.81 and J12.82; influenza pneumonia: J09.X1, J10.0, J10.00, J10.01, J10.08, J11.0, J11.00, and J11.08; other viral pneumonia: J12*; bacterial and other pneumonia: J13, J14, J15*, J16*, J17, and J18*; influenza disease: J09*, J10.1, J10.2, J10.8*, J11.1, J11.2, and J11.8*; acute respiratory distress syndrome: J80; chronic obstructive pulmonary disease with acute exacerbation: J44.1; asthma acute exacerbation: J45.21, J45.22, J45.31, J45.32, J45.41, J45.42, J45.51, J45.52, J45.901, and J45.902; respiratory failure: J96.0*, J96.2*, and R09.2; other acute lower respiratory tract infections: J20*, J21*, J22, J40, J44.0, J41*, J42, J43*, J47*, J85, J85.0, J85.1, J85.2, J85.3, and J86*; acute and chronic sinusitis: J01* and J32*; acute upper respiratory tract infections: J00*, J02*, J03*, J04*, J05*, and J06*; acute respiratory illness signs and symptoms: R04.2, R05, R05.1, R05.2, R05.4, R05.8, R05.9, R06.00, R06.02, R06.03, R06.1, R06.2, R06.8, R06.81, R06.82, R06.89, R07.1, R09.0*, R09.1, R09.2, R09.3, and R09.8*; acute febrile illness signs and symptoms: R50*, R50.81, and R68.83; acute nonrespiratory illness signs and symptoms: R19.7, R43*, R51*, R51.9, M79.1*, M79.10, M79.18, R65*, R53.81, R53.83, R57.9, R41.82, R40*, R53.1, R11.0, R11.10, R11.11, R11.15, R11.2, R21*, R10.0, R10.1*, R10.2, R10.3*, R10.8, R10.81*, R10.84, and R10.9. All ICD-10 codes with * include all child codes under the specific parent code.

§ Immunocompromising conditions were obtained from ICD-10 discharge codes. The specific codes used were Hematological Malignancy: C81.*, C82.*, C83.*, C84.*, C85.*, C86.*, C88.*, C90.*, C91.*, C92.*, C93.*, C94.*, C95.*, C96.*, D46.*, D61.0*, D61.2, D61.9, D70.0, and D71.*; Solid Malignancy: C00.*, C01.*, C02.*, C03.*, C04.*, C05.*, C06.*, C07.*, C08.*, C09.*, C10.*, C11.*, C12.*, C13.*, C14.*, C15.*, C16.*, C17.*, C18.*, C19.*, C20.*, C21.*, C22.*, C23.*, C24.*, C25.*, C26.*, C27.*, C28.*, C29.*, C30.*, C31.*, C32.*, C33.*, C34.*, C35.*, C36.*, C37.*, C38.*, C39.*, C40.*, C41.*, C42.*, C43.*, C44.*, C45.*, C46.*, C47.*, C48.*, C49.*, C50.*, C51.*, C52.*, C53.*, C54.*, C55.*, C56.*, C57.*, C58.*, C59.*, C60.*, C61.*, C62.*, C63.*, C64.*, C65.*, C66.*, C67.*, C68.*, C69.*, C70.*, C71.*, C72.*, C73.*, C74.*, C75.*, C76.*, C77.*, C78.*, C79.*, C7A.*, C7B.*, C80.*, D3A.*, Z51.0, Z51.1*, and C4A.*; Transplant: T86.0, T86.1, T86.2, T86.3, T86.4, T86.5, T86.81, T86.85, D47.Z1, Z48.2.*, Z94.*, and Z98.85; rheumatologic/inflammatory disorders: D86.*, E85, E85.1, E85.2, E85.3, E85.4, E85.8*, E85.9, G35.*, J67.9.*, L40.54, L40.59, L93.0.*, L93.2.*, L94.*, M05.*, M06.*, M07.*, M08.*, M30.*, M31.3*, M31.5*, M32.*, M33.*, M34.*, M35.3*, M35.8*, M35.9*, M46.*, and T78.40*; Other intrinsic immune condition of immunodeficiency: D27.9, D72.89, D80.*, D81, D81.0, D81.1, D81.2, D81.4, D81.5, D81.6, D81.7, D81.8*, D81.9, D82.*, D83.*, D84.*, D87.89, D89, D89.0, D89.1, D89.3, D89.4*, D89.8*, D89.9, K70.3*, K70.4*, K72.*, K74.3, K74.4, K74.5, K74.6, N04.*, R18.0; HIV: B20.*, B21.*, B22.*, B23.*, B24.*, B97.35, O98.7*, and Z21*. All ICD-10 codes with * include all child codes under the specific parent code.

¶ The index date for each hospitalization was defined as either the date of collection of a respiratory specimen associated with the most recent positive or negative SARS-CoV-2 test result before the hospital admission or the admission date (if testing occurred only after the admission).

** On April 19, 2023, CDC authorized an additional bivalent vaccine dose for adults aged ≥65 years and additional doses for persons who are immunocompromised. https://www.cdc.gov/media/releases/2023/s0419-covid-vaccines.html

†† Death was identified at each individual site and was defined as a death while hospitalized or ≤28 days after hospital admission.

§§ For VE against critical illness, case-patients were persons admitted to an ICU or who experienced in-hospital death associated with COVID-19, and control patients were persons hospitalized without COVID-19.

¶¶ 45 C.F.R. part 46.102(l)(2), 21 C.F.R. part 56; 42 U.S.C. Sect. 241(d); 5 U.S.C. Sect. 552a; 44 U.S.C. Sect. 3501 et seq.

***
https://covid.cdc.gov/covid-data-tracker/#vaccinations_vacc-people-booster-percent-pop5

†††
https://www.cdc.gov/vaccines/acip/meetings/downloads/slides-2022-09-01/08-COVID-Oliver-508.pdf

§§§
https://www.cdc.gov/vaccines/acip/meetings/downloads/slides-2023-04-19/06-COVID-Oliver-508.pdf
